# 357. High Rate of Adverse Events in Patients Receiving Oral Antimicrobials Under a COpAT Monitoring Protocol

**DOI:** 10.1093/ofid/ofad500.428

**Published:** 2023-11-27

**Authors:** Ciara Sawey, Colleen Burgoyne, Angelina Winbush, Craig Temple, Brandon W Qualls, Sonal Munsiff, Alexandra Yamshchikov

**Affiliations:** University of Rochester, San Carlos, California; Univ. of Rochester, Rochester, New York; School of Medicine and Dentistry, University of Rochester Medical Center, Rochester, New York; University of Rochester, San Carlos, California; University of Rochester Medical Center, Rochester, New York; Univ. of Rochester, Rochester, New York; University of Rochester School of Medicine and Dentistry, Rochester, New York

## Abstract

**Background:**

Oral linezolid (LZD), trimethoprim sulfamethoxazole (SXT), voriconazole (VRC), and itraconazole (ITC) are used often for prolonged durations and are associated with an array of adverse events (AEs). Establishing specific protocols for monitoring such courses of Complex Outpatient Antibiotic Therapy (COpAT) regimens as parenteral alternatives is a priority.

**Methods:**

Patients receiving LZD, SXT, VRC, ITC between January 1^st^, 2019 and December 31^st^, 2022 with monitoring by our Outpatient Parenteral Antimicrobial Therapy (OPAT) program staff were identified for retrospective review of electronic health records. Weekly labs were highly encouraged in the first month of therapy, and at least monthly thereafter. All patients were reviewed during weekly OPAT rounds. An AE grading tool was derived using Common Terminology Criteria for Adverse Events (CTCAE 2017) to capture common AEs associated with study drugs. Primary study endpoint was occurrence of up to three AEs of any severity during antibiotic therapy. Secondary analyses evaluated risk factors and treatment outcomes associated with AE occurrence using chi-square statistic (MedCalc Software Ltd.).

**Results:**

A total of 133 adverse events were identified in 70 (56.5%) of 124 patients receiving COpAT with LZD, SXT, VRC, ITC. Most common infections were pulmonary, bone/joint, and bacteremia/endocarditis (Figure 1). Median length of therapy ranged 21 days (LZD) to 102 days (ITC). Median time to AE onset ranged 8 days (SXT) to 14 days (VRC). Most AEs (88.3%) were < Grade 2 in severity, but 44 (62.9%) of 70 patients incurred > 2 AEs per treatment course. Although VRC incurred highest proportion of AEs, rates were > 50% for all agents (Figure 2), and gastrointestinal AEs were most common (Figure 3). Occurrence of AE of any severity was significantly associated with modification of drug regimen and change in treatment duration.

**Figure 1.**
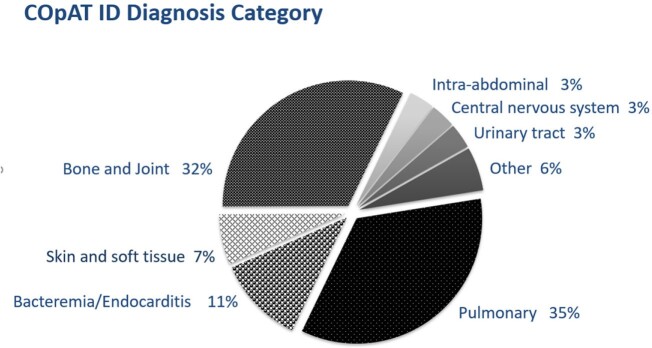
Infectious Disease Diagnoses Represented Under COpAT. Pulmonary, bone/joint, and endovascular infectious were most commonly represented.

**Figure 2.**
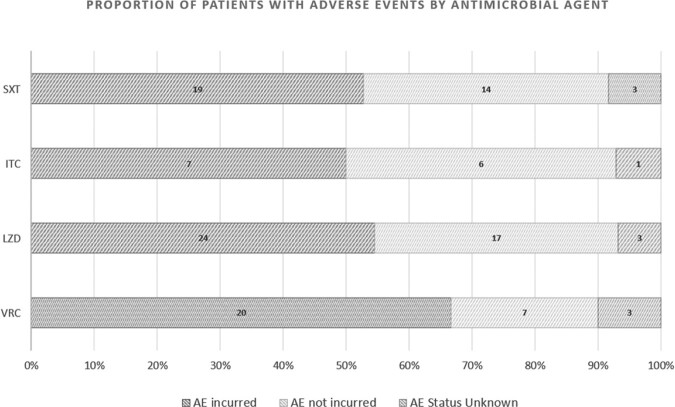
Proportion of Study Patients with Adverse Events by Medication. Adverse events were incurred in half or more of all treatment courses represented in this study

**Figure 3.**
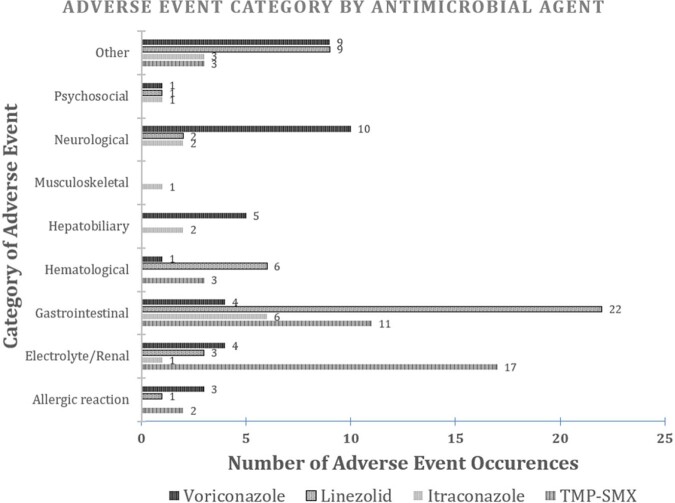
Adverse Event Categories by Medication.

Gastrointestinal toxicity was most frequently represented, followed by derangements in electrolyte or renal function.

**Conclusion:**

High rates of multiple low- grade AEs per patient treated were associated with use of oral LZD, SXT, VRC, and ITC for severe infections, and frequently required modifications in regimen or duration of therapy. Evidence based protocols are needed to help guide monitoring and clinical management for patients receiving COpAT with these important agents.

**Disclosures:**

**All Authors**: No reported disclosures

